# Newly generated cells are increased in hippocampus of adult mice lacking a serine protease inhibitor

**DOI:** 10.1186/1471-2202-11-70

**Published:** 2010-06-08

**Authors:** Maddalena M Lino, Catherine Vaillant, Slobodanka Orolicki, Melanie Sticker, Mirna Kvajo, Denis Monard

**Affiliations:** 1 Friedrich Miescher Institute for Biomedical Research, CH-4058 Basel, Switzerland; 2 Psychiatric Institute, Department of Psychiatry, Columbia University, New York, NY 10032, USA; 3 Novartis Pharma AG, Klybeckstrasse 141, CH-4057 Basel, Switzerland

## Abstract

**Background:**

Neurogenesis in the hippocampal dentate gyrus and the subventricular zone occurs throughout the life of mammals and newly generated neurons can integrate functionally into established neuronal circuits. Neurogenesis levels in the dentate gyrus are modulated by changes in the environment (enrichment, exercise), hippocampal-dependent tasks, NMDA receptor (NMDAR) activity, sonic hedgehog (SHH) and/or other factors.

****Results**:**

previously, we showed that Protease Nexin-1 (PN-1), a potent serine protease inhibitor, regulates the NMDAR availability and activity as well as SHH signaling. Compared with wild-type (WT), we detected a significant increase in BrdU-labeled cells in the dentate gyrus of mice lacking PN-1 (PN-1 ^-/-^) both in controls and after running exercise. *Patched homologue 1 *(*Ptc1*) and *Gli1 *mRNA levels were higher and *Gli3 *down-regulated in mutant mice under standard conditions and to a lesser extent after running exercise. However, the number of surviving BrdU-positive cells did not differ between WT and PN-1 -/- animals. NMDAR availability was altered in the hippocampus of mutant animals after exercise.

****Conclusion**:**

All together our results indicate that PN-1 controls progenitors proliferation through an effect on the SHH pathway and suggest an influence of the serpin on the survival of newly generated neurons through modulation of NMDAR availability.

## Background

Neurogenesis occurs throughout the life of mammals [1,2] where the hippocampal dentate gyrus and the subventricular zone (SVZ) retain the ability to generate new neurons during adulthood [3,4]. In the hippocampus, granule neurons are generated from a population of continuously dividing cells residing in the subgranular zone of the dentate gyrus [2,5,6]. These "newborn" progenitor cells migrate into the granule cell layer, differentiate, extend axons and express neuronal marker proteins [7].

Newly generated neurons can integrate functionally into neuronal circuits [8] and represent a powerful means of brain repair. Neurogenesis in the dentate gyrus can be modulated by enrichment of the environment and by behavior, such as exercise and hippocampal-dependent tasks [4,9-11]. In particular, voluntary exercise in a running wheel has been shown to be the most efficient mean of increasing hippocampal cell proliferation, cell survival and net neurogenesis [11-13]. In contrast, exposure to acute psychosocial stress results in rapid decline of proliferation in the dentate gyrus [14,15].

At present, little is known about the mechanisms controlling the generation of new neurons. Neuron generation and survival can be mediated partially by trophic factors [16] such as brain-derived nerve growth factor (BDNF) [17-20], vascular endothelial growth factor [21], insulin like growth factor [22], fibroblast growth factor [23], SHH [24] and others. A further mechanism implicated in adult brain neurogenesis is excitatory input and NMDAR activation. Blockade of NMDAR increases proliferation in the dentate gyrus [14,15] and the overall density of neurons in the granule cell layer [25]. Moreover, it was reported recently that survival of new neurons is regulated by the relative levels of NMDAR activation [26,27].

Previously, we showed that the serine protease inhibitor PN-1 regulates NMDAR availability, leading to an altered electrophysiology detected so far in the hippocampus and the barrel cortex [28,29]. Recently we found that PN-1 contributes to shaping of the cerebellum by promoting cell cycle exit through inhibition of SHH signaling [30].

During embryogenesis and in the postnatal brain, PN-1 is expressed prominently at different times in areas of high remodeling [31,32]. In particular, the distributions of *Shh *and *PN-1 *transcripts overlap in various developing organs. In the developing central nervous system, *Shh *and *PN-1 *are co-expressed in the ventral part of the mesencephalon and myelencephalon, the mid-hindbrain junction, cerebellum and optic vesicles [32]. Recently, we showed that PN-1 modulates SHH signalling strength during postnatal development of the cerebellum in mice. In particular, in *PN-1 *deficient mice, the proliferation of the granular cells neuronal precursors is increased while initiation of their differentiation is delayed. This results in overproduction of mature granular cells and subsequent expansion of regionalized lobes [30]. It was therefore of great interest to investigate whether adult neurogenesis, especially cell proliferation and survival, is affected in the hippocampus of mice lacking PN-1.

## Results

### PN-1 expression in the dentate gyrus

**Figure 1:**
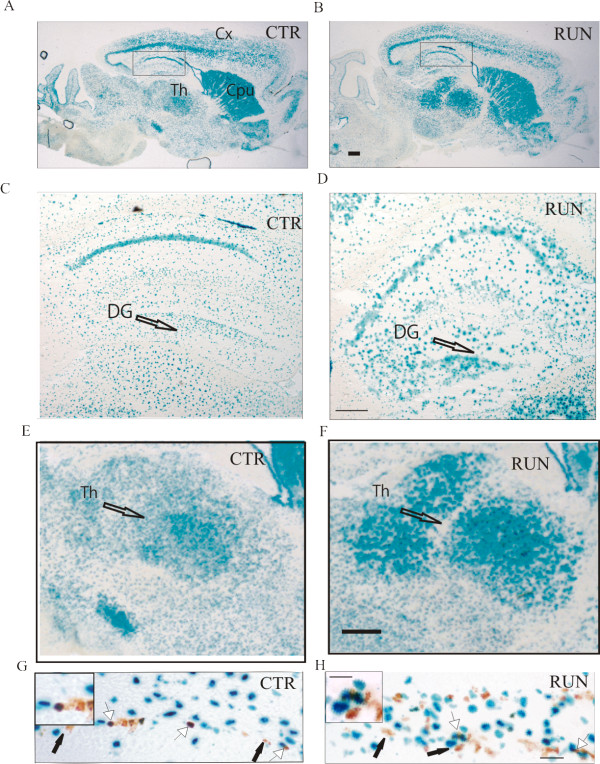
**PN-1 expression increases after running wheel exercise**. (*A-B*) PN-1 expression detected by X-Gal staining in the brain of a reporter knock-in mouse (PN-1KI) without (CTR) and after 12 days of running exercise (RUN). (*C, D*) Enlargement of the dentate gyrus in control and exercised animals, respectively. (*E, F*) Enlargement of the thalamus in control and exercise animals, respectively. (*G, H*) BrdU and X-Gal staining in controls and after running, respectively. BrdU-positive cells (brown) lack PN-1 (black arrows) or are positive for PN-1 (white arrows). Enlargements are shown on the upper left. Cx: Cortex; DG: Dentate gyrus; Th: Thalamus. Scale bars: *A, B, G *and *H *800 μm (insert in H 400 μm), *C, D *400 μm, *E, F *100 μm.

Using PN-1 knock-in reporter mice (PN-1 KI) [29], we first analyzed PN-1 expression by monitoring X-Gal staining in the brain following running wheel exercise (Fig. 1). In the basic situation, PN-1 was strongly expressed in the cortex, caudato-putamen, thalamus, lateral ventricles, CA1 field and dentate gyrus of the hippocampus. After 12 days of exercise, a marked increase in PN-1 expression was evident in the thalamus and dentate gyrus (Fig. 1A-D). Progenitor cell proliferation in the dentate gyrus was estimated by bromodeoxyuridine (BrdU) labeling of dividing cells over 12 days. Cell proliferation increased in the dentate gyrus after running and X-Gal/BrdU double labeling revealed few newborn cells being both BrdU and PN-1 positive (Fig. 1E, F).

### Cell proliferation in WT and PN-1 ^-/- ^mice

**Figure 2:**
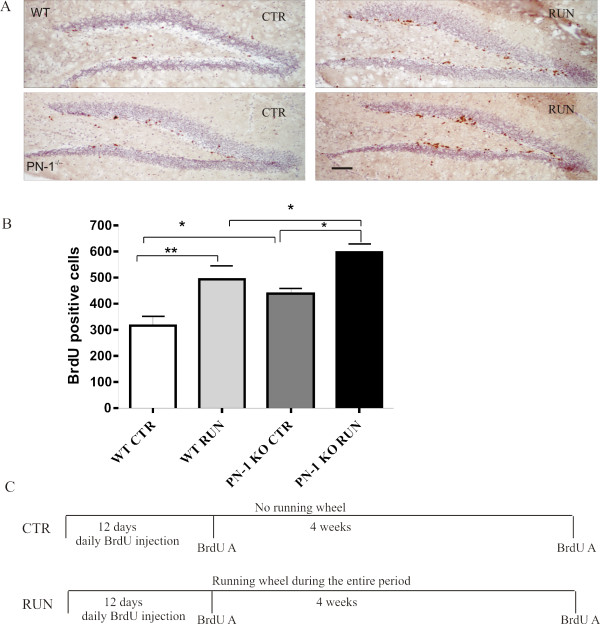
**PN-1 ^-/- ^mice show increased BrdU incorporation in the dentate gyrus**. (*A*) BrdU-positive cells in dentate gyrus sections of WT and mutant mice without and with 12 days of running wheel exercise. (*B*) Number of BrdU-positive cells in WT and PN-1 ^-/- ^mice without and after running. All data are the means of 4 animals/condition/genotype, *P < 0.05, **P < 0.01. Scale bar: *A *100 μm. (C) Scheme illustrating the timeline of the running injection protocol. WT and PN 1^-/- ^mice kept without and with running wheel, were injected with BrdU daily for 12 days. BrdU analysis was performed either one day after the last BrdU injection or 4 weeks later. BrdU A: BrdU analysis.

A systematic study revealed a significant difference in the number of BrdU-labeled cells in WT littermates and PN-1-deficient mice (Fig. 2A-B). Already in the control non-challenged situation, there was a significant increase (+26.6%) in proliferating cells in the dentate gyrus of mice lacking PN-1 (Fig. 2B). The difference was still evident at the same level (+23%) after 12 days of running exercise (Fig. 2B). Thus, lack of PN-1 expression promotes proliferation in both control and running conditions. The distance covered during the experimental period did not significantly differ between WT and PN-1-deficient mice. During the running period no special behavioral alterations have been noticed in the PN-1 KO animals. A 3-D reconstruction, to search for an increase in size affecting a specific substructure of the dentate gyrus, was performed 12 days after running as described [30]. No size differences were detected between WT and PN-1 lacking mice (results not shown).

### SHH pathway activation is influenced by PN-1 expression and running exercise

**Figure 3:**
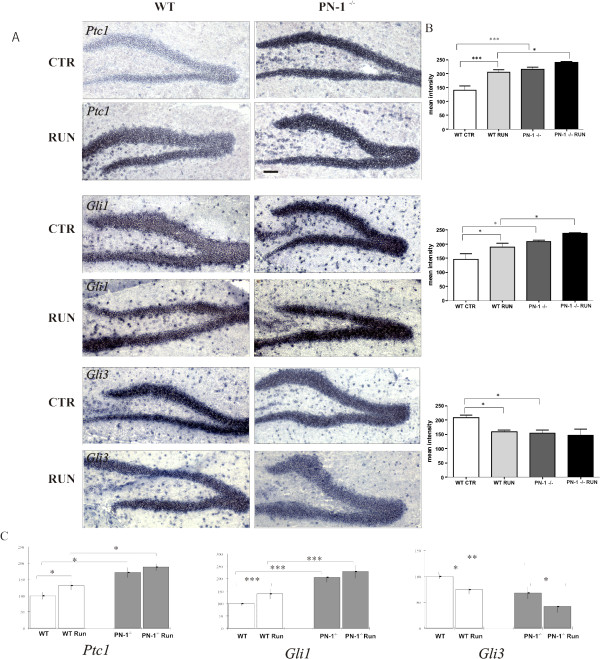
**Increase of *Gli1 *and *Ptc1 *and decrease of *Gli3 *expression in PN-1 ^-/- ^mice**. (*A*) *In situ *hybridization of *Ptc1, Gli1 *and *Gli3 *in WT and PN-1 ^-/- ^mice without (CTR) and with 12 days of running wheel exercise (RUN). (*B*) Quantification of the *in situ *hybridization of *Ptc1, Gli1 *and *Gli3 *in WT and PN-1 ^-/- ^mice without and with 12 days of running wheel exercise (*C*) Changes in *Ptc1, Gli1*, and *Gli3 *expression detected by RT-PCR in wild type and mutant mice without and with running exercise. All data are the means of 3 animals/condition/genotype. *P < 0.05, **P < 0.01; ***P < 0.001. Scale bar: *A *100 μm.

Blocking the SHH pathway with cyclopamine leads to reduced cell proliferation in the dentate gyrus, while overexpression of SHH produces the opposite effect [24]. Given the increase in PN-1 expression in the dentate gyrus after running exercise (Fig. 1) and the known influence of PN-1 on SHH-mediated cell proliferation in the cerebellum [30], we evaluated SHH signaling by monitoring the expression of *Patched 1 *(*Ptc1*) and of the transcriptional regulators *Gli1 *and *Gli3 *by *in situ *hybridization and RT-PCR. *Ptc1 *and *Gli1 *expression was higher and *Gli3 *lower in PN-1 ^-/- ^mice under standard conditions (Fig. 3A). After 12 days of exercise, *Ptc1 *and G*li1 *mRNA levels had increased in WT littermates and to a lesser extent in PN-1 ^-/- ^animals. Exercise led to a decrease in *Gli3 *expression in WT mice (Fig. 3A). These results were confirmed by quantification of the images (Fig. 3B). In addition RT-PCR analysis of the entire hippocampus provided similar results, even detecting the reduction of *Gli3 *upon running in mutant animals (Fig. 3C). No difference in the level of *Shh *expression was detected in WT and mutant mice (data not shown). Altogether, these data show that SHH pathway activation is modulated by PN-1 expression and running exercise.

### Survival of newly generated neurons is impaired in PN-1 ^-/- ^mice

**Figure 4:**
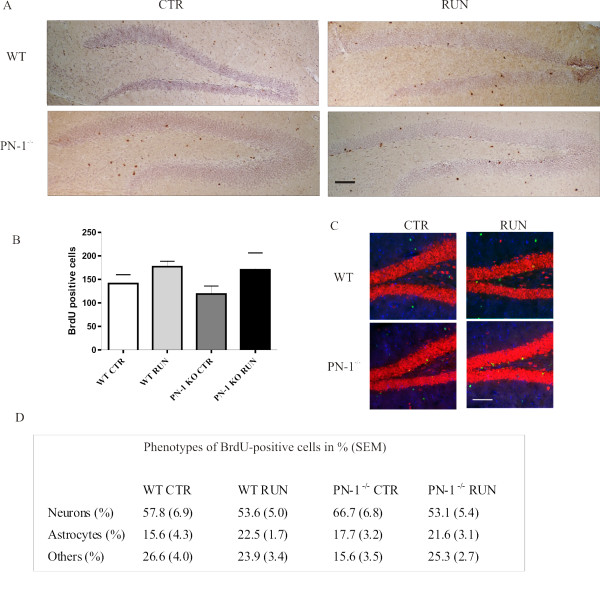
**Reduced survival of Brdu positive cells generated by running exercise in PN-1 ^-/- ^dentate gyrus**. (*A*) Number of BrdU-positive cells (brown) in dentate gyrus 4 weeks after the end of BrdU injection in WT and PN-1 ^-/- ^mice without (CTR) and with running wheel exercise (RUN). (*B*) Lack of differences in the number of BrdU-positive cells in WT and PN-1 ^-/- ^mice without and with exercise. (C) Imaging of double labeled BrdU/NeuN and BrdU/S100 cells in hippocampus of WT and PN-1 KO mice with and without running condition. BrdU: green (Alexa 488), NeuN: red (Alexa 594), S100: violet-cy5 (Jackson 633). (*D*) Phenotypes of surviving cells determined by immunofluorescent triple-labeling for BrdU, NeuN (neurons) and S100 (astrocytes). The percentages of BrdU-positive cells double-labeled for either S100 or NeuN are shown. All data are the means of 3 animals/condition/genotype.

Survival and differentiation of the progeny of dividing progenitor cells were monitored 4 weeks after the last BrdU injection (Fig. 3). The total number of surviving BrdU-positive cells was the same in WT littermates and PN-1 ^-/- ^mice, although an excess of newly proliferating cells was detected in mutant mice (Fig. 4B versus Fig. 2B). The survival rates, calculated as the ratio of BrdU-positive cells after 4 weeks to BrdU-positive cells after 12 days of running exercise, were different in WT and mutant mice, namely 40% to 27% for the control unchallenged animals and 39% to 30% for the animals which had been running. There was, thus, a decline in the survival of newly generated cells in the PN-1 ^-/- ^mice. The differentiation of these cells was evaluated four weeks after the last BrdU injection by triple labeling with BrdU, the neuronal marker NeuN and the glial marker S100. The ratio of cell phenotypes did not differ significantly between the WT and PN-1 ^-/- ^animals (Fig. 4C). In conclusion, the excess of newly produced cells observed in PN-1 ^-/- ^mice does not affect hippocampal cytoarchitecture suggesting a lack of survival.

### Different effects of running on NMDAR subunit expression in wt vs. PN-1 -/- animals

Given that NMDAR availability and function are reduced in the hippocampus and the barrel cortex of PN-1 ^-/- ^mice [28,29], we were intrigued by the reports that receptor subunits availability changes after running challenge [9,15,33,34]. Moreover, it was recently reported that NMDAR is needed for the integration of new neurons in the adult dentate gyrus [26,27].

**Figure 5:**
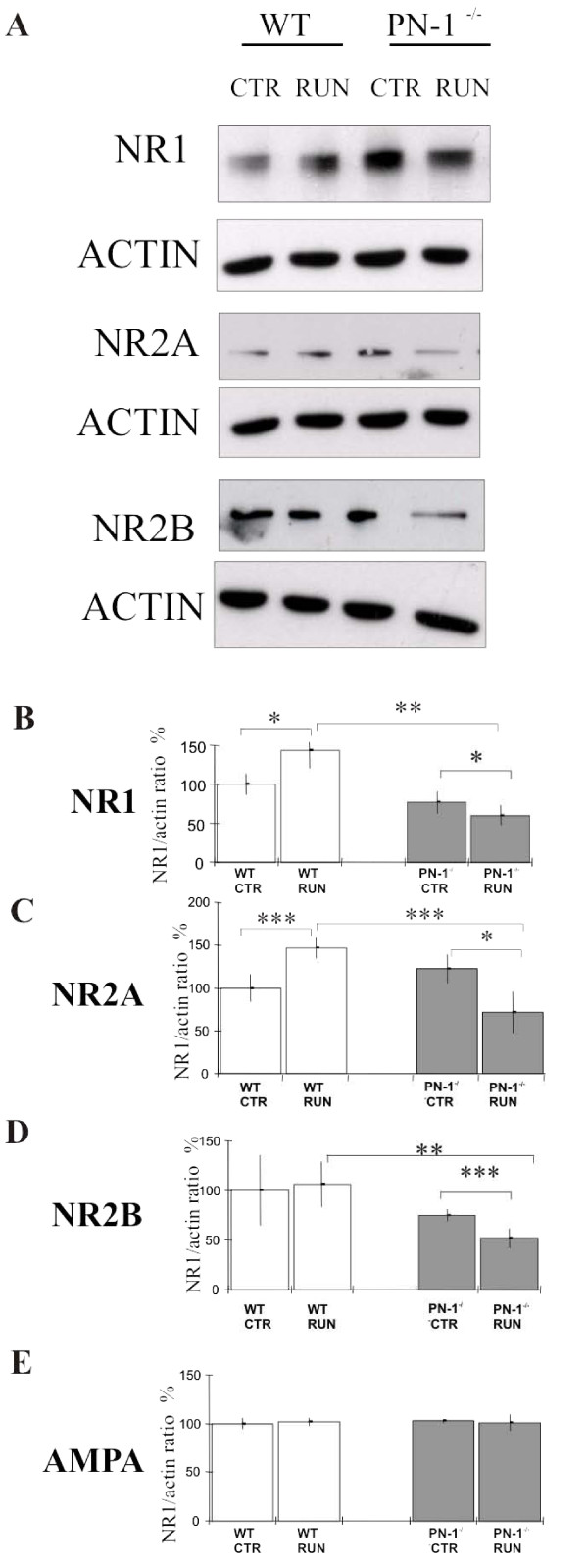
**Effect of running exercise on NMDAR availability in wild type and PN-1 ^-/- ^mice**. (*A*) Representative immunoblot detecting NR1, NR2A and NR2B NMDAR subunits in hippocampal synaptosomal fraction of WT and PN-1 ^-/- ^mice without (CTR) and with running exercise (RUN). (*B-E*) Quantification of similar immunoblots including AMPA receptor as control. All data are the means of 3 animals. For each treatment, values in the WT without exercise were taken as 100% relative to the actin level.

Consequently, we estimated the NMDAR subunits expression levels by Western blot analysis in synaptosomal fractions from the hippocampus of WT and mutant mice under normal and running conditions. Similar levels of NMDAR NR1 subunits were detected in PN-1 ^-/- ^and WT littermates housed under standard unchallenged conditions. After 12 days of running exercise, NR-1 immunoreactivity increased in WT mice but a significant reduction was observed in PN-1 ^-/- ^animals (Fig. 5A, B). The NR2A but not the NR2B subunit increased in WT littermates with exercise. Both subunits decreased in PN-1 ^-/- ^mice after 12 days of running activity (Fig. 5A, C, D). Levels of alpha-amino-3-hydroxy-5-methyl-4-isoxazolepropionic acid (AMPA) receptor were similar in the WT and mutant animals and did not change following exercise (Fig. 5E), indicating a specific effect of PN-1 on NMDAR.

## Discussion

Our results show that mice deficient for the endogenous protease inhibitor PN-1 have a higher proliferation rate in the hippocampus. However, the newly produced cells do not survive. The increased proliferation rate is mainly associated with a constitutive overactivation of the SHH pathway and abnormal NMDAR availability could account for the altered survival.

SHH is one of the critical regulators of neurogenesis, including proliferation of adult hippocampal neural stem cells *in vitro *and *in vivo *[24,35]. The importance of the SHH signaling pathway has been demonstrated using cyclopamine or ectopic expression of the protein [24]. These authors showed that SHH is not expressed locally in the hippocampus, but anterogradly transported from the basal forebrain via the fornix. Similar to the situation in the developing cerebellum [30], the increased *Gli1 *and *Ptc1 *expression and the decreased *Gli3 *mRNA levels observed here in the hippocampus of PN-1 ^-/- ^mice (Fig. 2) indicate an enhancement of the SHH signaling pathway. Our results show for the first time that running exercise triggers the hippocampal SHH pathway. The stimulation is weaker in PN-1 ^-/- ^than in WT mice. This is probably due to a high preexisting basal level of SHH signaling evidenced by increased *Gli1 *and *Ptc1*, respectively decreased *Gli3 *mRNA levels in the mutant mice.

We cannot exclude that the lower availability of NMDAR detected in mutant mice after running could also contribute to an increase in progenitor cell proliferation. Similar to the responses to enriched environment [29], 12 days of running exercise led to a decrease in the levels of NR1, NR2A and NR2B NMDAR subunits in PN-1 ^-/- ^mice (Fig. 4 A-D). The impact of NMDAR stimulation on hippocampal cell proliferation is still a matter of debate. NMDAR antagonism has been shown to stimulate adult neurogenesis in the dentate gyrus [15,33,34,36], while a recent study supports a stimulatory function upon short-term NMDA signal transduction [36]. The decline in the availability of NMDAR subunits in the mutant mice after running may be functionally similar to the antagonism of NMDAR, thus contributing to the detected increase in proliferation. In fact, as the increase in the already enhanced SHH pathway after exercise is quite marginal in the mutant mice, the impact due to reduced NMDAR activation could be more important in these animals.

We investigated whether the higher number of cells produced in the mutant would lead to an increase in cells able to survive and integrate. In fact, only a small fraction of the newly generated cell population survived in the adult brain. For example, in the hippocampus or the SVZ, 50% of the newborn cells double-labeled with neuron-specific markers [37] were found to die within 2 months of birth [5,10,38-40]. This phenomenon is enhanced in PN-1 ^-/- ^mice. The difference in the number of BrdU-positive cells in WT and mutant mice found after 12 days, both with and without exercise (Fig. 2A, B), was not observed 4 weeks later (Fig. 2C,D). Moreover, a 3-D reconstruction did not provide evidence for an increase in size affecting a specific substructure of the dentate gyrus, as detected in the cerebellum of PN-1 ^-/- ^mice [30]. The excess of progenitor cells resulting from PN-1 gene deficiency is probably neutralized by altered survival.

This observation is in line with the proposal that NMDAR activation promotes neuronal differentiation and integration [26,27,34,36]. In particular, the NR2B subunit was identified recently as one of the major players in the functionality of newly generated neurons [41]. It is thus interesting to stress that this subunit decreased significantly in the PN-1 mutant following running exercise (Fig. 4D). The reduction may thus explain the observed decrease in neuronal integration in the mutant. Consequently, the failure of PN-1 ^-/- ^mice to integrate cells generated during the first 12 days of exercise may also be due to the reduced NMDA-dependent stimulation detected in the cortex and hippocampus of the mutant mice [28,29].

## Conclusion

The results from this study reveal a dual effect of PN-1, which regulates SHH-induced proliferation and may also sustain NMDAR-controlled neuronal integration. In summary, our data identify a PN-1 modulatory function at the crossroad of the SHH and NMDAR signaling pathways, the interplay of which coordinates proliferation versus survival and integration in a neurogenic territory.

## Methods

### Animals

Adult WT, knock-in and PN-1^-/- ^mice [28] were divided into four groups: WT and PN-1^-/- ^mice runners and their controls. The construct used for the knock-in mice (PN-1KI) allows independent translation of PN-1 and β-galactosidase from the transcript [29]. The controls were housed under standard conditions with 3-4 mice per cage. The runners (3-4 animals per cage) were housed for 12 days in a standard cage with one Linton exercise wheel activity counter (model EWAC-R). A different set of animal under the same conditions was used to study NMDAR subunits. C57BL/6 mice were purchased from Charles River (Arbresle, France). All experimental animals were 3 months old.

### BrdU injection

BrdU (Sigma, St Louis, Missouri) was dissolved in 0.9% Nacl and filtered sterile at 0.2 μm. The mice received single doses of 50 μg/g body weight through one daily intraperitoneal injection of a 10 mg/ml solution, always at the same time for 12 consecutive days [10]. All animal experiments were approved by the Swiss Veterinary Authorities.

### Immunohistochemistry

For BrdU immunohistochemistry and immunofluorescent triple labeling for BrdU, NeuN and S100b, perfused brains were post-fixed for 12 h in 4% PFA, equilibrated for 24 h in 30% sucrose and quickly frozen in Tissue-Tek O.C.T (Sakura Finetek, USA). Cryosections of 20 μm were mounted on slides to give a series of 6 slides with comparative sections. Prior to antibody incubation, sections were treated in a microwave processing lab-station (Milestone) with citrate buffer, pH 6 for 10 min at 97°C. The primary antibody treatments and the controls were incubated at 4°C. The antibodies used were rat anti-BrdU (Immunologicals Direct, Oxford, UK) 1:300, mouse anti-NeuN (Chemicon, Temecula/CA, USA) 1:1000 and rabbit anti-S100b (Swant, Bellinzona, Switzerland) 1:5000. To detect BrdU-labeled cells. biotinylated goat anti-rat antibody (Vector Laboratories, Burlingham/CA, USA) was applied, followed by the ABC-kit (Vector) and DAB as a substrate. The fluorescent secondary antibodies were goat anti-rat Alexa 488, goat anti-mouse Alexa 594 (Molecular Probes) and goat anti-rabbit Cy5 (Jackson ImmunoResearch). For counting BrdU and triple labeled positive cells we used a Nikon Eclipse E600 microscope equipped with a Leica camera DFC420 with 10 × and 20 × objective, respectively.

### Protein analysis

Synaptosomal-enriched plasma membrane were prepared as described earlier [29]. Immunoblotting was performed using SDS PAGE and the NuPAGE protein detection systems (Invitrogen) according to the manufacturer's instructions. The antibodies used were anti-NR1 (1:1000) and anti-NR2B (1:500) (UPSTATE Charlottesville, Va.), anti-NR2A (1:500) (Santa Cruz, Santa Cruz, Calif., USA) and anti-actin (1:5000) (NeoMarkers, Fremont, Calif., USA).

### β-galactosidase immunohistochemistry

PN-1 Knock-in (PN-1 KI) mice [29] were divided into running and control experimental groups as described above. β-galactosidase immunohistochemistry was performed as described earlier [29].

### Statistical analysis

One 20-μm section out of four throughout the whole hippocampus were stained and BrdU-positive cells counted on 12 sections covering the entire dentate gyrus. One-way ANOVA with Newman Keul's multiple comparison tests was used for the statistical analysis. Statistical analysis of RT-PCR was performed by *t*-test assuming unequal variances. Groups of three to four animals were used for each experiment.

### RT-PCR

Total RNA was prepared using the RNeasy kit (Qiagen) according to the manufacturer's instructions. First-strand cDNA was synthesized using AMV Reverse Transcriptase (Promega) according to the manufacturer's instructions. Aliquots (2 μl) of each cDNA was amplified by PCR with the following primers: *Shh*: forward: 5'gctgctgctggccagatg 3'; reverse: 5'gttcggagtttcttgagatc 3'; *Gli1*: forward: 5'tgccagatatgcttcagcca 3', reverse: 5'acctctgtgtctattcgccac 3'; *Ptc1*: 5'ccaaactccactcaaaaggtgc 3', 5'cattggcaggaggagttgattg 3'; *Gli3*: forward: 5'gtgccatcgatgaaacc 3'; reverse: 5'ctacgatcccatctccacag 3'. β-*actin *PCR products were used to normalize the results (forward: 5'gtgggccgctctaggcacaa 3' and reverse: 5'ctctttgatgtcacgcacgatttc 3'). Bands were visualized and quantified by Gene Snap software (Syngene). Thirty cycles were used for *Shh*, *Gli1 *and *Gli3 *detection, 32 cycles were used for *Ptc1 *and 28 cycles were used for actin detection.

### In situ Hybridization

*In situ *hybridization was performed using 10-μm sagittal brain sections. *Gli1, Gli3 and Ptc1 *mRNA probes were as described [42]. Three animals per genotype and conditions were used for each experiment. Image-ProPlus sofware was used for the quantification of the *in situ *signals.

## Abbreviations

BDNF: brain-derived nerve growth factor; CTR: Control; NMDAR: NMDA receptor; *Ptc1*: *Patched homologue 1*; PN-1: Protease Nexin-1; SHH: sonic hedgehog; SVZ: subventricular zone.

## Authors' contributions

ML assisted in the study conceptualization, design and coordination and carried out the histological and molecular assays and drafted the manuscript. CV performed the 3 D analysis and participated in the manuscript write up. MK participated in the design study and in the discussion during the all study. SO performed the NMDA receptor study. MS performed part of the histological staining. DM assisted the study, its coordination and its design and led the manuscript write up.
